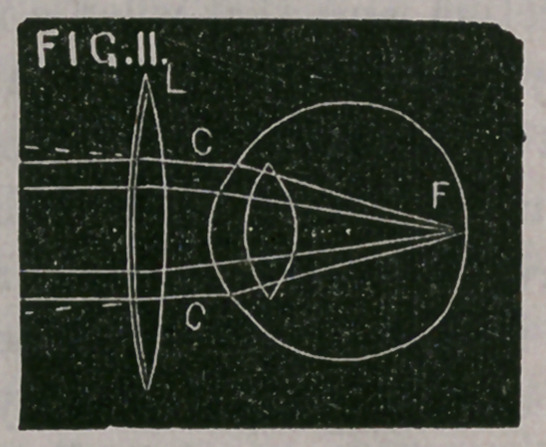# An Introduction to the Study of the Optical Defects of the Eye and Their Treatment by the Scientific Use of Spectacles

**Published:** 1866

**Authors:** A. M. Rosebrugh

**Affiliations:** Toronto, C. W.


### AN INTRODUCTION TO THE STUDY OF THE OPTICAL DEFECTS OF THE EYE AND THEIR TREATMENT BY THE SCIENTIFIC USE OF SPECTACLES.

(Continued from No. 7, page 270.)

BY A. M. ROSEBRUGH, M. D., TORONTO, C. W.

### Chapter III—Myopia.

*Concave Lenses.*—Before proceeding to the consideration of myopia, it will be well for us to glance at some of the properties of concave lenses; and in order to simplify the subject, we will confine ourselves to equi-concave lenses. An equi-concave lens is bounded by two surfaces, which are portions of the concave side of two circles which have equal radii.

Fig. 9. A, B, one of the concave surfaces of the lens. C is the centre of curvature, and C, R the radius of curvature. When parallel rays, P, P, strike one surface of the lens, they have a divergence upon leaving the second surface of the lens, as if they proceeded from the centre of curvature, C, which, in an equi-concave lens, is also the principal focus of the lens. C, R, is the focal length of the lens. In a convex lens, the focus is measured *behind* the lens; in a concave lens, it is measured *in front* of it. If we call the focus of the convex lens positive, we must call the focus of the concave lens negative. When parallel rays of light fall upon a convex lens, they are converged to a focus. When they fall upon a concave lens, they are made to diverge. A convex lens enlarges, and a concave diminishes the apparent size of objects. The focal length of a convex lens is measured behind; and that of a concave lens, in front of the lens. They are, therefore, entirely opposite in all their properties; and, for this reason, a convex lens is called a positive lens; and a concave one, a negative lens. Or, shorter still, they are indicated by the plus (+) and minus (—), algebraic symbols; thus, + 5, and — 5; or, + $\frac{1}{5}$, and — $\frac{1}{5}$. To ascertain the focal length of a concave lens, we ascertain what convex lens it will neutralize.

1. In a myopic eye, parallel rays, as well as those that have a certain degree of divergence, are focussed *in front* of the retina; and, the inverted image of distant objects being formed in the same position, the picture upon the retina will be ill-defined, and vision for distant object consequently indistinct.

Patients with myopia complain that, although their vision for near objects is perfect, they cannot see objects at a distance with any distinctness. They can read the smallest type, when brought near the eyes, even better than persons with normal vision, but they are not able to recognize their friends at a distance of fifteen or twenty feet.

In order to enable such persons to see distinctly at a distance, it is necessary for them to wear concave spectacles of such a strength, that the parallel rays from distant objects may have such a degree of divergence, that, falling upon the myopic eye, they may form a focus upon the retina. Theoretically, we should prescribe concave glasses of such a strength that their focus will correspond with the patient's "far" point. Thus, if the "far" point be at 12 inches, we should prescribe — 12, as a twelve inch concave lens, placed before such an eye, will give parallel rays from distant objects the same degree of divergence as if they proceeded from the "far" point of the eye; namely, at 12 inches from the eye. Thus, in Fig. 9, P. P. represent parallel rays falling upon the concave lens, A. B.; they are made to diverge, as if coming from the focus, C., and falling upon the eye divergingly, they are focussed upon the retina at F. Practically, however, we would find that — 12 would be rather too strong, and that — 15, or — 16 would probably answer better. As a rule, the weakest glasses should be worn that will enable the patient to see distant objects with distinctness.

In testing the degree of myopia, we use a series of test types that are so constructed that No. I (smallest) can be distinctly seen and read by a person having normal vision, at a distance of 1 foot; No. II, at 2 feet; No. V, at 5 feet; No. XX, at 20 feet; and so on. A specimen of these types will be annexed to this paper. The types are also used in testing the acuteness of vision in Presbyopia, Hypermetropia, Amblyopia, &c.

2. In determining the degree of myopia in any case, we ascertain the greatest distance at which No. I test types can be read distinctly; if at 10 inches, the "far" point will be at 10 inches, and the myopia would be called $\frac{1}{10}$; if at 6 inches, the myopia would be called $\frac{1}{6}$. From this we can, as stated above, get a proximate knowledge of the strength of the concave lens necessary to relieve the myopia.

3. A myopic eye, when in a state of rest, is adjusted for diverging rays. To enable such an eye to see distant objects, that is, to bring parallel rays to a focus on the retina, it is necessary to give these parallel rays a preliminary degree of divergence by the interposition of the proper concave lens.

4. Myopia can be distinguished from every other defect of vision, by the fact that concave glasses improve vision for distant objects. If we have no concave glasses convenient, we can diagnose it from Amphyopia, (insensibility of the retina) by the following ready method:—A person with normal vision can read distinctly, No. I test type at 12 inches, and even a little farther. We will suppose that a patient's vision is so impaired, that he can only read No. II at 6 inches; if he is *not* also myopic, he can also read No. IV at 12 inches, or No. LX at 180 inches—that is at 15 feet. However impaired then a person's vision may be, unless he be also myopic, he can see as well proportionately, at one distance as at another. On the contrary, a person with myopia, say $\frac{1}{6}$, can see the smallest type (much smaller than No. I,) at 6 inches, but he cannot see No. II, or even No. V, at 12 inches.

This disease is often hereditary. Over exertion of the eyes upon near objects at the age of puberty, (about 14 or 15) is a very frequent cause of myopia.

Short-sighted persons often inquire if we would advise the use of spectacles. There can be no objection to wearing glasses that will enable them to see distant objects; for their eyes are thus changed to normal ones, but as most persons use their eyes much more frequently upon near than upon distant objects; the glasses should be no stronger than necessary. Some contend, however, that short-sighted persons should dispense with glasses for reading, writing, &c. Prof. Donders, however, recommends their use for this purpose, for the following reasons:—

1st. "Because strong convergence of the optic axes is necessarily paired with tension of the accommodation. The latter is an associated action, not arising from the mechanism of the convergence, but existing within the eye itself, and may consequently easily lead to an increase of the myopia. Besides this, the pressure of the muscles upon the eye ball appears to be greater when the optic axes are convergent, than when they are parallel, and this increase of pressure cannot but tend to give rise to the development of posterior staphyloma.

2d. "On account of tho habit which short-sighted persons have of bending their head forwards during reading or writing. This must cause an increased flow of blood to the eye, and an increased tension within the eye itself. Owing to this development of sclerotico—choroiditis posterior, effusions of blood and detachment of the retina which are so apt to occur in short-sighted persons, are undoubtedly greatly promoted. For this reason, we should always tell these patients to read with their head well thrown back, and to write at a sloping desk. But it may, on the other hand, be urged that it is just in looking at near objects that myopic persons have an advantage, for they can see them remarkably distinctly. And the great danger is, that after reading for a short time with spectacles, the patient, on getting somewhat fatigued will, instead of laying the book aside, approach it nearer to the eye, in order to gain greater retinal images, and thus strain and tax his power of accommodation too much. If we, for instance, give a patient whose far point lies at 8 inches, a pair of spectacles which enable him to read at 12 inches, he will, if not very careful, after a short time almost insensibly bring the book nearer to his eyes, and thus have to make use of a greater amount of accommodation. If he does this frequently, he will soon increase his myopia. The greater the range of accommodation the less harm will spectacles do, and *vice versâ*. Spectacles may also be used for near objects in those cases of myopia in which asthenopia (depending upon insufficiency of the internal recti muscles) shows itself as soon as the patient has read or worked at near objects for a short time. Whilst these forms of myopia may be furnished with spectacles for near objects, it is very dangerous to permit their use in patients whose range of accommodation is very limited, and who, moreover, suffer perhaps from such an amount of amblyopia (generally depending upon sclerotico—choroiditis posterior) that they cannot read No. 4 or 5 Jäger even with the most accurately chosen glasses. Such patients will bring the object very close to the eye, in order to obtain large retinal images, the accommodation will be greatly strained, the intra-ocular tension be increased, and great mischief will be sure to ensue. If there is much amblyopia, spectacles should not be permitted at all for near objects."*

In cases where the myopia is extreme, there usually co-exists posterior staphyloma of the sclerotic. Von Græfe says it is present in all cases of myopia where the "far" point is less than five inches; the myopia being less than $\frac{1}{5}$. Out of sixty cases of myopia examined by J. Z. Laurence, forty-four had posterior staphyloma.

The presence of this disease can be easily diagnosed with the ophthalmoscope. (See Hulke or Zander on the ophthalmoscope.)

*Mr. J. Z. Laurence, of London, recommends that deeply concave lenses be *tinted*, in order to obviate their "dazzling" effect.—(Med. Times and Gazette, Oct. 22nd, 1864.)

Posterior staphyloma is a serious complication in myopia, as the sensibility of the retina becomes more or less impaired in the position of the bulging of the sclerotic, and in some cases the retina becomes detached from the choroid. It is the existence of this disease that prevents improvement in cases of myopia, as the eye becomes flattened with advancing age.

Donders considers that in myopia, the antero-posterior diameter is alone at fault; that is, it is too much elongated, and that the cornea and crystaline lens have usually a normal curvature.

The characteristics of a myopic eye, are*

1st. Parallel rays are focussed in front of the retina.

2nd. The "far" point is at a definite distance and positive.

3rd. When the eye is in a state of rest it is adapted for divergent rays.

4th. Concave glasses improve vision.

### Chapter IV.—Hypermetropia.

You will remember that when a normal eye is in a state of rest, and directed to a distant object, parallel rays are brought to a focus upon the retina, and that when a myopic eye is in a state of rest, parallel rays are brought to a focus in front of the retina. When, however, a hyperemtropic eye is in a state of rest, parallel rays would (if continued) form a focus behind the retina. Hypermetropia is, therefore, the reverse of myopia. In myopia, the refractive power of the eye is excessive, and in hypermetropia it is not strong enough. When the accommodation of a myopic eye is paralysed, it has the power of focussing none but diverging rays upon the retina, but a hypermetropic eye under the same circumstances can focus only converging rays upon the retina. The "far" point of a myopic eye is at a definite distance and positive, but the "far" point of a hypermetropic eye is at a definite distance and negative. Concave glasses improve the vision for a myopic eye, and convex for a hypermotropic one.

This is an affection which has received very little attention until within the last ten years. It was indeed noticed by Dr. McKenzie of Glasgow, in 1841, but it was not until about five years ago that

* From Donders' system of classification.

Prof. Donders, of Utrecht, from his elaborate researches on this subject, first pointed out how common this affection is, and how frequently it is the sole cause of that peculiar weakness of sight (formerly so little understood) called asthenopia.

Donders believes that this condition of the eye depends more upon a shortening of the antero-posterior diameter of the eye, than upon a too low degree of its refractive power; that the cornea and crystaline lens have a normal degree of curvature, and that parallel rays would form a focus at the normal distance behind the lens, were the retina far enough back to receive it.

A very good illustration of a hypermetropie eye is one in which the crystaline lens has been removed in the operation for cataract. To enable such an eye to see distinctly, even distant objects, it is necessary to place in front of it a strong convex lens of about four inches focus, called a cataract glass. The eye having too low a refractive power to converge rays to a focus, on the retina, it is necessary to give rays falling upon the eye, a preliminary degree of convergence; the eye having sufficient power to complete their refraction to a point upon the retina. We do the same thing in relieving cases of hypermetrophia.

Fig. 10 represents a hypermetropie eye in a state of rest. P P are parallel rays which are focussed behind the retina at f. L, Fig. 11, is a convex lens which changes the parallel rays to convergent ones, at c, c, as if they came from the direction *a b* and *d e*, which again are refracted by the eye, and brought to a focus upon the retina at F.

When a hypermetropie eye is in a state of rest, and directed to distant objects, it is adjusted for convergent rays; images upon the retina will consequently be ill defined, and vision will be indistinct. To remedy this, it is necessary for the eye to increase its refractive power by increasing the antero-posterio diameter of the crystaline lens, so as to bring parallel rays to a focus on the retina.

When a person with hypermetropia, attempts to read or write, or accommodate his eyes to short distances, it is necessary for him to tax his accommodation to its utmost extent, in order to bring the diverging rays to a focus on the retina. This excessive effort at accommodating the eye for short distances, can not be kept up for more than a few minutes, when the ciliary muscle begins to relax,—the "near" point commences to receed, and (if he is reading) the letters become indistinct. The eye also feels fatigued, and other symptoms arise which will be referred to when speaking of Asthenopia.

*Diagnosis.*—When we suspect a patient has hypermetropia, we test his eyes as follows :—We place a series of test-types, No. xv., xx., xxx., &c., at a distance of about 20 feet. If he can read No. xv. or xx. at this distance, his acuteness of vision is normal. We then try his vision with weak convex glasses, say No. 50, and if he can read the same type, at the same distance, we try successively No. 40, 36, 30, 24, &c., until we reach the glasses that render the test type indistinct at that distance. Some persons may possibly be able to relax their accommodation so as to see as well at a distance, with convex 50 lenses, as without them; and not be hypermetropic; it would, however, be very strong presumptive evidence of its presence; and if, in addition, the patient complain of the symptoms of Asthenopia, we would be generally safe in pronouncing it a case of hypermetropia. The shorter the focus of the lens he can use, the stronger is the presumptive evidence of the disease.

Again, if another patient be tested with the same type, at the same distance, and we find that he can not read a smaller type than No. XL. at 20 feet without spectacles, and that he can read No. xv. or xx. with convex glasses, say + 10 or + 12, his would be called a case of hypermetropia *absolute.*

In order, however, to test accurately the degree of hypermetropia in any case, it is necessary to neutralize one element in the refractive power of the eye; namely, the power of accommodation. In most cases of hypermetropia, particularly in young subjects, the accommodation of the eye is so constantly exercised, even when directed to distant objects, that it is quite impossible for them, by any effort of their own, to completely relax that accommodation. I related in a former chapter, the case of a patient who had lost the power of accommodating his eye to different distances. As the refraction of his eye was normal, parallel rays were brought to a focus upon the retina, and vision for distant objects remained perfect.

Had his eye been hypermetropic, parallel rays would not have been sufficiently converged by the refractive power of the eye, to form a focus upon the retina; vision would, consequently, have been indistinct. By placing, however, the proper convex lens in front of such an eye, the requisite preliminary convergence would be given to the rays, to enable the eye, with its low refractive power, to focus these rays upon the retina, and thus render vision distinct.

The lens used in such a case would indicate the degree of hypermetropia. If the lens were a + 15 inch, the hypermetropia would equal $\frac{1}{15}$ if a + 10, the hypermetropia would be $\frac{1}{10}$, and so forth.

We have, however, the means of temporarily producing this condition of the eye by artificial means. By applying a four grain solution of atropine to the eye, within two hours the action of the ciliary muscle will be completely paralysed. A solution of one grain of atropine to an ounce of pure water (also a solution of the extract of belladonna) will dilate the pupil widely, and in some cases, will render the eye slightly presbyopic, but it will not paralyse the accommodation.

If we test, in this manner, the case of suspected hypermetropia mentioned above, and find that after his accommodation is paralysed, he is not able to read No. xxx. even with + 50, and that the only glass with which he can read No. xv. and No. xx. at 20 feet is + 20; his hypermetropia is therefore $\frac{1}{20}$. But as he could see as well with + 50 as without them, before his accommodation was paralysed; he had a manifest hypermetropia of $\frac{1}{50}$. The difference between his total hypermetropia and his manifest hypermetropia will give the amount of the *latent* hypermetropia, which he overcame with the exercise of his accommodation, namely, $\frac{1}{33\frac{1}{3}}$, thus $\frac{1}{20} - \frac{1}{50} = \frac{1}{33\frac{1}{3}}$.*

*Asthenopia*, according to Donders, depends almost invariably on hypermetropia. He describes it as follows: "The power of vision is usually acute,—and nevertheless, in reading, writing, and other close work, especially by artificial light, or in a gloomy place, the objects after a short time, become indistinct and confused, and a feeling of fatigue and tension comes on in, and especially above the eyes, necessitating a suspension of work. The person affected now often involuntarily closes his eyes, and rubs his hand over the forehead and

* Hypermetropia can easily be diagnosed with the ophthalmoscope.

eyelids. After some moments rest, he once more sees distinctly, but the same phenomena are again developed more rapidly than before."

According to my own experience with these cases, the above description corresponds very closely with the description that most patients give of their symptoms. Some give more prominence to the neuralgic pains which they experience in and around the eye, and in some cases extending to the back of the head. I was consulted, about a year ago, by a lady from the town of Simcoe, C.W., who had all these symptoms in the most aggravated form. If she attempted to read even one line, it gave her so much pain in her eyes and forehead that, for several years, she had scarcely dared to even raise the lid of a book. She was unable to keep her eyes upon any one object for more than an instant at a time, without causing her pain. Others, again, do not speak of any pain or fatigue of the eye; but that, after reading a short time, the letters become indistinct, so that they are obliged to stop or look away at something distant, or close the eyes for a short time, when they can again proceed, the same symptoms recurring.

In regard to the *prognosis* in hypermitropia, Donders thinks that when it is once developed it never gives way. All the inconvenience of the accompanying Asthenopia can be relieved by wearing the proper glasses to relieve the hypermetropia; but the cause, namely (in most cases), a congenital flattening of the eye-ball from before, backwards, will probably remain through life.

As age advances, the "near" point recedes from the eye, as in a normal eye, so that in time it becomes complicated with presbyopia.

*Treatment.*—In order to correct this optical defect, it is necessary for the patient to wear a pair of convex spectacles of sufficient strength to enable him to see distant objects distinctly, without any effort of the accomodation. In cases where the hypermetropia is absolute, and the patients are not able to see distinctly at any distance, they can, approximately, by trial, select the glasses that will remedy the low degree of refraction of their eyes. But, in all other cases, it is necessary to paralyse the accomodation, and test with lenses of different strength, in order accurately to ascertain the degree of hypermetropia. When we ascertain this fact, we also know the number of the glasses that we must prescribe for them. The effect of the atropine usually lasts about a week, after which the patient can commence wearing glasses. Before, however, he use the spectacles that he is to wear permanently, his accomodation must first be gradually relaxed by the use of weaker lenses. Donders' rule is to prescribe first that glass that will neutralize his manifest hypermetropia, and $\frac{1}{4}$ of his latent hypermetropia, and every two or three weeks change them for a stronger pair, as he becomes accustomed to their use, until the glasses are reached that we found to be necessary to correct his hypermetropia. Thus, if a patient has a total amount of hypermetropia equal to $\frac{1}{10}$, and a manifest hypermetropia of $\frac{1}{30}$, his latent hypermetropia ($\frac{1}{10} - \frac{1}{30} = \frac{1}{15}$), would equal $\frac{1}{15}$; one fourth of $\frac{1}{15}$ is $\frac{1}{60}$; this, added to $\frac{1}{30}$ ($\frac{1}{30} + \frac{1}{60} = \frac{3}{60} = \frac{1}{20}$), equals $\frac{1}{20}$. We would therefore prescribe, at first, 20 inch convex spectacles, which we would afterwards change successively for + 18, + 16, + 14, &c., until he has so relaxed his accomodation that he can, with ease, wear + 10. It will not be until he becomes accustomed to this last pair that all his symptoms of Asthenopia will disappear.

*Strabismus.*—Prof Donders was the first to direct attentlon to the fact, that nearly all cases of convergent strabismus arise from the presence of hypermetropia. We know that when both eyes are directed to a near object, they are very much converged,—the optic axes cross at the point to which they are directed. If one eye be covered, and the opposite eye be accommodated for its "near" point, the convered eye will be found to be very decidedly converged towards the nose,—to have, in fact, a temporary convergent squint. This arises from the constant association of the act of accommodating the eye for short distances, with the act of contracting the internal recti muscles. The hypermetropic, however, being obliged to exert the accommodation of their eyes, even when looking at distant objects, it is easy to understand that they would be inclined to contract their internal recti-muscles unduly, so as to increase this power of accommodation. This converges the eyes to a point at a nearer distance than the object looked at, and causes one ot the eyes to turn inwards, while the other is fixed upon the object. When, therefore, they wish to see distinctly with one eye, they instinctively turn in the other. At first the convergent strabismus is seen occasionally only, and in this stage may be prevented by using the proper spectacles to correct the hypermetrophia. After the squint has existed sometime, it becomes confirmed and cannot be cured without an operation.

If the convergence exceeds three lines, a partial tenotomy, upon each eye, should be performed, and the effect controlled by a conjunctival suture, by which means we have the power of regulating our operation, in proportion to the effect we wish to produce.

When Strabismus shows itself in childhood, it should be treated without delay, for, if not corrected, the vision of the "cross-eye" will very soon become impaired.

To get the full benefit of spectacles, in cases of hypermetropia, they should be used both on the street, and at church, as well as when reading or writing,—in fact whenever the eyes are used.

The characteristics of a hypermetropic eye then are :

1st. Parallel rays form a focus behind the retina.

2nd. The "far" point is at an definite distance and negative.

3rd. The eye, in a state of rost, is adjusted for convergent rays.

4th. Convex glasses improve vision.

5th. This affection is usually accompanied by symptoms of Asthenopia and Amblyopia, and frequently by convergent strabismus.

### Chapter V.—Presbyopia.

This affection usually develops itself between the ages of 40 and 45. Most persons at this age, although previously enjoying excellent vision, complain that their sight, particularly in the evening, is beginning to fail for near objects, as small print, &c., although they can see distant objects as well as ever.

In reading they will hold the book or paper at nearly arm's length and perhaps bring the lamp almost between their eyes and the page. Reading in this manner soon fatigues them, and they are obliged frequently to rest,—or to resort to spectacles.

In childhood, when the vision is normal, the "near" point is from 3½ to 4 inches from the eye, and the "far" point at an unlimited distance ; that is, we can see objects distinctly as near as from 3½ to 4 inches from the eye, and we can see objects clearly (the size being in proportion to the distance) from that to an indefinite distance. As age advances the "near" point recedes. At the age of 40 the "near" point is about eight inches from the eyes. When the "near" point recedes to a greater distance than 8 inches, Donders calls it a case of presbyopia ; Laurence, however, thinks that it should not be called presbyopia unless the "near" point is at least 10 inches from the eye.

Presbyopia, then, is not an optical defect of the nature of myopia or hypermetropia, but is simply a lessening of the accommodative power of the eye.

It is supposed to depend upon, or to be caused by, the crystaline lens becoming hardened as age advances, so that it does not yield sufficiently to the contraction of the ciliary muscle.

In a case of pure presbyopia where, for instance, the "near" point is 12 inches from the eye, vision will remain normal for all points beyond that distance. When the "near" point is 12 inches distant, and the "far" point at an infinite distance, the accommodation is only $\frac{1}{12}$. Taking eight inches as the normal "near" point, $\frac{1}{8}$ would represent the normal accommodation. Deducting $\frac{1}{12}$ frem $\frac{1}{8}$ gives the degree of presbyopia thus: $\frac{1}{8} - \frac{1}{12} = \frac{1}{24}$. The degree of presbyopia in this case would then be $\frac{1}{24}$. This fraction $\frac{1}{24}$ also represents the strength of the glasses necessary to correct the presbyopia, namely 24 inch convex. Practically, we would probably find that a pair of 30 inch convex would answer better, as the weakest glass that can be worn with comfort, is the one that should be prescribed. Again, if a person's "near" point be at 16 inches, his presbyopia ($\frac{1}{8}-\frac{1}{16}=\frac{1}{16}$) will be $\frac{1}{16}$, and a 16 inch convex lens would enable him to read at 8 inches.

"There can be no question as to the advisability and necessity of affording far-sighted persons the use of spectacles. They should be furnished with them as soon as they are in the slightest degree annoyed or inconvenienced by the presbyopia. Some medical men think that presbyopic patients should do without spectacles as long as possible, for fear the eye should, even at an early period, get so used to them as soon to find them indispensable. This is, however, an error, for if such persons are permitted to work without glasses, we observe that the presbyopia soon rapidly increases."*

If, however, we call all cases presbyopia, where the "near" point recedes to a greater distance than eight inches from the eye, it will follow that we may have presbyopia in cases of myopia and hypermetropia. If a person's far point be at 20 inches from the eye he would be called *near-sighted* and if his near point recedes to 10 inches from the eye, he would be also *far-sighted*.

In some persons, as age advances, the "far" point also recedes so

* J. Soelberg Wells.

as to render the person hypermetropic ; this form of hypermetropia seldom exceeds $\frac{1}{24}$. When a person has both hypermetropia and prebyopia, it is necessary for him to use a stronger pair of glasses for reading, &c., than for ordinary use. If a person for instance, wears a pair of 18 inch convex spectacles to correct a hypermetropia of $\frac{1}{18}$, and as age advances his "near" point recedes to 12 inches, even with the addition of his glasses, it will be necessary for him to wear, for reading, a pair of glasses having a focus of about $10\frac{1}{2}$ inches. Thus $\frac{1}{8} - \frac{1}{12} = \frac{1}{24}$ = presbyopia, this added to the lens to correct his hypermetropia, ($\frac{1}{18} + \frac{1}{24} = \frac{1}{10\frac{1}{2}}$ nearly) equals $10\frac{1}{2}$ nearly.

In the very aged, it is necessary to prescribe glasses, that will enable them to read at 5 or 7 inches from the eye, as their vision is usually somewhat impaired.

The following table constructed by Dr. Kitchener may give a general idea of the glasses required at different periods of life when the presbyopia is unaccompanied by hypermetropia or amblyopia.

| | | | | | | | |
|---|---|---|---|---|---|---|---|
| At 40 | years,— | 36 | inch focus. | At 70 | years,— | 12 | inch focus. |
| " 45 | " | 30 | " " | " 75 | " | 10 | " " |
| " 50 | " | 24 | " " | " 80 | " | 9 | " " |
| " 55 | " | 20 | " " | " 85 | " | 8 | " " |
| " 58 | " | 18 | " " | " 90 | " | 7 | " " |
| " 60 | " | 16 | " " | " 100 | " | 6 | " " |
| " 65 | " | 14 | " " | | | | |

Prof. Donders thinks that when there is no hypermetropia present we should generally advise those glasses to be worn that will enable the person to read distinctly No. I (smallest) test type at a distance of 12 inches.

There is an optical defect of the eye that is occasionally met with called astigmatism (from *a* and *στίγμα*) in which horizontal and vertical lines are not brought to a focus at the same distance behind the crystaline lens. It is relieved by glasses specially ground for each case, these glasses are cylindrical. I have seen but one case of astigmatism.

A very comprehensive article on this subject appears in the Medical Times and Gazette, Nov., 1864, from the pen of J. Zachariah Laurence, M.B., of London.

The paralysis of the accommodation of the eye I have already referred to in a case on page 268.

## Figures and Tables

**FIG. 9. f1:**
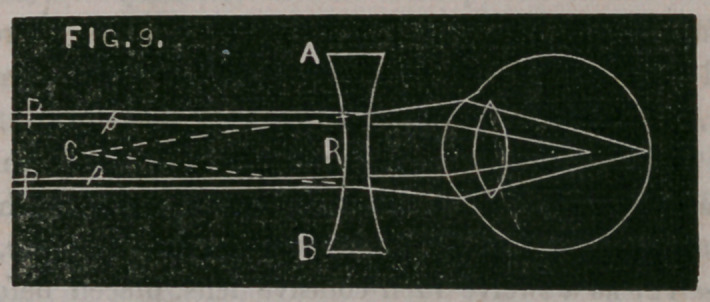


**FIG. 10. f2:**
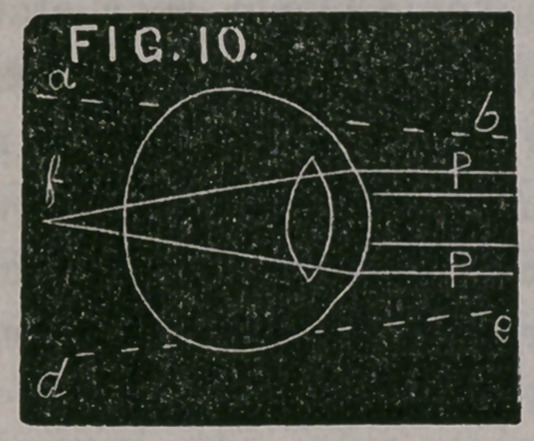


**FIG. 11. f3:**